# Complex executive functions assessed by the trail making test (TMT) part B improve more than those assessed by the TMT part A or digit span backward task during vagus nerve stimulation in patients with drug-resistant epilepsy

**DOI:** 10.3389/fpsyt.2024.1349201

**Published:** 2024-02-14

**Authors:** Niina Lähde, Pabitra Basnyat, Jani Raitanen, Leena Kämppi, Kai Lehtimäki, Eija Rosti-Otajärvi, Jukka Peltola

**Affiliations:** ^1^Department of Neurology, Tampere University Hospital, Tampere, Finland; ^2^Faculty of Medicine and Health Technology, Tampere University, Tampere, Finland; ^3^Faculty of Social Sciences, Health Sciences, Tampere University, Tampere, Finland; ^4^UKK Institute for Health Promotion Research, Tampere, Finland; ^5^Epilepsia Helsinki, Member of EpiCARE ERN, Department of Neurology, Helsinki University Hospital and University of Helsinki, Helsinki, Finland; ^6^Department of Neurosurgery, Tampere University Hospital, Tampere, Finland; ^7^Department of Rehabilitation and Psychosocial Support, Tampere University Hospital, Tampere, Finland

**Keywords:** attention and executive functions, digit span backward, drug-resistant epilepsy, processing speed, set-switching, trail-making test, vagus nerve stimulation, working memory

## Abstract

**Introduction:**

There is a paucity of clinical studies examining the long-term effects of vagus nerve stimulation (VNS) on cognition, although a recent study of patients with drug-resistant epilepsy (DRE) treated with VNS therapy demonstrated significant improvement in executive functions as measured by the EpiTrack composite score. The present study aimed to investigate performance variability in three cognitive tests assessing executive functions and working memory in a cohort of DRE patients receiving VNS therapy during a follow-up duration of up to 5 years.

**Methods:**

The study included 46 DRE patients who were assessed with the Trail Making Test (TMT) (Parts A and B) and Digit Span Backward (DB) task prior to VNS implantation, 6 months and 12 months after implantation, and yearly thereafter as a part of the clinical VNS protocol. A linear mixed-effects (LME) model was used to analyze changes in test z scores over time, accounting for variations in follow-up duration when predicting changes over 5 years. Additionally, we conducted descriptive analyses to illustrate individual changes.

**Results:**

On average, TMT-A z scores improved by 0.024 units (95% confidence interval (CI): 0.006 to 0.042, *p* = 0.009), TMT-B z scores by 0.034 units (95% CI: 0.012 to 0.057, *p* = 0.003), and DB z scores by 0.019 units per month (95% CI: 0.011 to 0.028, *p* < 0.001). Patients with psychiatric comorbidities achieved the greatest improvements in TMT-B and DB z scores among all groups (0.0058 units/month, *p* = 0.036 and 0.028 units/month, *p* = 0.003, respectively). TMT-A z scores improved the most in patients taking 1–2 ASMs as well as in patients with psychiatric comorbidities (0.042 units/month, *p* = 0.002 and *p* = 0.003, respectively).

**Conclusion:**

Performance in all three tests improved at the group level during the follow-up period, with the most robust improvement observed in TMT-B, which requires inhibition control and set-switching in addition to the visuoperceptual processing speed that is crucial in TMT-A and working-memory performance that is essential in DB. Moreover, the improvement in TMT-B was further enhanced if the patient had psychiatric comorbidities.

## 1 Introduction

Vagus nerve stimulation (VNS) is an adjunctive treatment option for patients with drug-resistant epilepsy (DRE) and refractory depression. VNS has demonstrated efficacy for seizure control in randomized controlled trials (1, 2), long-term extension studies (3, 4) and real-world follow-up studies (5, 6). Recently, there has been increasing interest in the effects of VNS on cognition since cognitive impairment is a common comorbidity in both DRE (7) and depression (8). Working memory performance and executive functions are particularly impaired in patients with epilepsy (9–11). Moreover, up to 75% of DRE patients may have deficits in attention and executive functions (AEF) (12).

To date, most of the research conducted on VNS and cognition has focused on memory, with limited coverage of other cognitive functions. Existing data suggest potential improvements in working memory, visual attention, and verbal memory performance following VNS therapy (13–17). However, there remains a paucity of clinical studies, especially those examining the long-term effects of VNS on cognition. Notably, a recent study demonstrated significant improvement in AEF among DRE patients receiving VNS therapy with a follow-up period of up to 5 years (18).

The Trail-Making Test (TMT) is one of the most common tests for evaluating executive functions and has been demonstrated to be useful in assessing executive functions of epilepsy patients in previous studies (19, 20). The TMT consists of two parts; the TMT-A provides a baseline measure of psychomotor speed, visuospatial search, and target-directed motor tracking (21), while the TMT-B is similar to the TMT-A in assessing low-level processes, but it additionally measures other more advanced components of executive functions, such as inhibition control and set-switching (22–24). In addition to these high-level AEFs, the TMT-B task also requires working memory, whereas the Digit Span Backward (DB) task is primarily used to exclusively assess working memory (21). Interestingly, a previous computer-based study of DRE patients treated with VNS demonstrated an improvement in working memory performance due to the direct effects of VNS (14).

Accumulating data suggest that all these three tests (TMT-A/B and DB) are associated with brain networks susceptible to the direct effects of VNS. Functional magnetic resonance imaging (fMRI) studies have indicated that TMT performance is mediated by large-scale brain networks, including prefrontal and parietal structures associated with the default mode network (DMN) (25). Moreover, the neural substrates linked with number sequence recitation in the DB test have been identified in the intraparietal sulcus and perisylvian areas, both of which are connected to the vagus afferent network (VAN). In turn, the VAN is directly and indirectly connected to the DMN (26).

Having observed significant improvement in AEF among DRE patients receiving VNS therapy in our recent study (18) as measured by the EpiTrack (27) composite score, we aimed to delve deeper into the diverse effects of VNS on individual tests (TMT-A, TMT-B and DB) included in the EpiTrack and linked to VAN. Consequently, our study focused on assessing the performance in repeated TMT-A, TMT-B and DB tests over a follow-up period of up to 5 years in a group of DRE patients treated with VNS therapy.

## 2 Materials and methods

### 2.1 Study design

This was a noninterventional study in which data were collected prospectively but analyzed retrospectively from a VNS quality register at Tampere University Hospital; thus, ethics committee approval was not required, according to the Finnish Law on Research. Access to the VNS quality register was granted by the Tampere University Hospital Research, Development and Innovation Centre. This manuscript adheres to the applicable Strengthening the Reporting of Observational Studies in Epidemiology (STROBE) statement.

### 2.2 Patients

This study included 46 DRE patients who were implanted with VNS (Model 106 (Aspire®), Model 1,000 (SenTiva®) or Model 102) at Tampere University Hospital and evaluated with the TMT and DB prior to and after implantation with repeated follow-ups. For this study, all patients implanted with VNS from September 1, 2013, to February 28, 2021, with a minimum follow-up of 12 months until the end of February 2022, and at least two postimplantation assessments were included.

Patients with intellectual disabilities were excluded because they were unable to perform the tests. All patients had previously undergone a presurgical evaluation and were either unsuitable candidates for resective epilepsy surgery or had undergone surgery but did not achieve adequate seizure control.

### 2.3 Patient characteristics

We retrospectively extracted information on years of education, concomitant psychiatric comorbidity (either current or in the past), Beck Depression Inventory II (BDI-II) score at baseline, age at epilepsy onset, duration of epilepsy, etiology and type of epilepsy, predominant seizure type and frequency during the 12 months prior to VNS implantation and 3 months prior to each postimplantation assessment, current antiseizure medication (ASM) use, model and duration of VNS, and previous resective epilepsy surgery or other brain surgery from the VNS quality register.

Epilepsy type was classified as temporal lobe epilepsy (TLE), frontal lobe epilepsy (FLE), or other (one case of parietal lobe epilepsy; five cases of multilobar epilepsy including two frontotemporal, and one each of temporoparietal, parietofrontal, and temporo-occipital; four cases of multifocal epilepsy, and one case of unspecified genetic generalized epilepsy). The etiology of epilepsy was evaluated from MRI findings and clinical history. Classification of seizure type was determined by video-electroencephalogram (EEG) findings and seizure semiology. The predominant seizure type (focal aware seizure (FAS), focal impaired awareness seizure (FIAS), and focal to bilateral tonic–clonic seizure (FBTCS)) for each patient was defined as the most disabling seizure type noted in the medical records as determined by the physician, not necessarily the most frequent seizure type (28). Patients with FAS and FIAS were combined into a single group in the analysis. One patient was seizure-free at baseline (predominant seizure type FBTCS), and the frequency of the predominant seizure type was not available for one patient (FIAS). These two patients were excluded from the analysis of the effect of predominant seizure type on test performance. All patients were treated with ASMs (range 1 to 4) in addition to VNS, and two patients were concomitantly treated with deep brain stimulation (DBS) of the anterior nucleus of the thalamus (ANT). We defined ASM burden reduction as ASM withdrawal and/or dose reduction and ASM burden increase as ASM addition and/or dose increase during follow-up. The clinical characteristics of the patients are presented in Table 1.

**Table 1:** Demographics and clinical characteristics of the patients.

Total patients (*n* = 46)	Descriptives
**Age at baseline in years (median, (IQR))**	33 (28–43)
**Sex (female/male)**	23/23
**Educational years (median, (IQR))**	12 (12–14)
**Psychiatric comorbidity**	
*Yes (n, %)*	*12 (26.1)*
*Present/Past*	*7/5*
*No (n, %)*	*34 (73.9)*
**BDI at baseline (median, (IQR))**	5 (2–10)
**Age at epilepsy onset in years (median, (IQR))**	16 (10.8–23)
**Epilepsy duration in years (median, (IQR))**	15.5 (10–24)
**ILAE Etiology (*n*, %)**	
**Structural**	**15 (32.7)**
*Cortical developmental malformations*	*5 (10.8)*
*Vascular lesion*	*4 (8.7)*
*AV-malformation*	*2 (4.3)*
*Cavernoma*	*1 (2.2)*
*Brain trauma*	*1 (2.2)*
*Late effects of radiation*	*1 (2.2)*
*Hippocampal sclerosis*	*1 (2.2)*
**Immune**	**4 (8.7)**
*Limbic autoimmune encephalitis*	*4 (8.7)*
**Infectious**	**2 (4.3)**
**Genetic**	**1 (2.2)**
**Unknown**	**24 (52.2)**
**Epilepsy type (*n*, %)**	
*Frontal lobe epilepsy*	*18 (39.1)*
*Temporal lobe epilepsy*	*17 (37.0)*
*Other*	*11 (23.9)*
**Predominant seizure type at baseline (*n*, %)**	
*FAS*	*5 (11)*
*FIAS*	*29 (63)*
FBTCS^*^	*12 (26)*
**Number of ASMs at baseline (*n*, %)**	
*1*	*1 (2.2)*
*2*	*16 (34.8)*
*3*	*25 (54.3)*
*4*	*4 (8.7)*
**VNS model (*n*, %)**	
*1000 (Sentiva®)*	*14 (30.4)*
*106 (Aspire®)*	*25 (54.3)*
*102*	*7 (15.2)*
**Duration of VNS therapy in months (median, (range))**	31.5 (12–60)
**Previous brain surgery (resective or other)**	
*Yes (n, %)*	*9 (20)*
*No (n, %)*	*37 (80)*

^*^Including one patient with GTCS as the predominant seizure type. ASM = antiseizure medication, BDI = Beck’s Depression Inventory, FAS = focal aware seizure, FBTCS = focal to tonic clonic seizure, FIAS = focal impaired awareness seizure.

### 2.4 Cognitive evaluation

The patients were assessed with the TMT and DB test prior to VNS implantation, at six and 12 months after implantation, and yearly thereafter as a part of our standard clinical VNS protocol. These tests were performed either as a part of full neuropsychological evaluation or EpiTrack testing for VNS follow-up. In the TMT-A, the subject must connect encircled numbers 1 to 25 distributed on a sheet of paper in ascending order as quickly as possible without lifting the pen off the paper; Part B is similar, except alternation is between numbers and letters (1, A, 2, B, 3, C, etc.).

The time needed to complete the tasks was the analyzed variable (21). In the DB test, numbers are presented by the examiner, and subjects repeat the numbers in reverse order until they fail two times at a given level. The total number of correct answers was used in the analysis. The raw scores of the tests were converted into age-standardized z scores using published Finnish (29) and international normative data (30).

In this study, z scores of equal to or less than −3 were considered indicative of a severe deficit; z scores from −2.99 to −2 were indicative of a moderate deficit; z scores from −1.99 to −1 were indicative of a mild deficit, and z scores equal to or greater than −0.99 were considered to demonstrate normal performance. Moreover, clinically meaningful improvement in test performance was defined as an increase of more than one standard deviation (SD) in z scores or a change in performance category. The median duration of VNS/follow-up time after implantation was 31.5 months and ranged between 12 and 60 months. Furthermore, due to the COVID-19 pandemic, scheduled appointments did not always take place according to our protocol. Therefore, changes in the z scores over time were analyzed using a linear mixed-effects (LME) model to compensate for the variation in follow-up duration when predicting changes in the z scores over 5 years. The actual timing of the assessments is presented in Supplementary Figure S1.

### 2.5 Statistical analysis

Changes in TMT-A, TMT-B, and DB z scores over time (months) were analyzed using a multilevel mixed-effects linear regression (LME) model with robust standard errors in Stata version 17.0 (StataCorp, College Station, Texas, United States). The outcome variables were the average z scores (continuous) for each test, and the exposure variables were clinical characteristics (psychiatric comorbidities, epilepsy types, predominant seizure types, and ASMs) and time (continuous, in months). Visual representations of the results include observed values of the z scores for each test at each time point and fitted average trajectories based on LME models. In addition, the changes in the z scores for each test over a follow-up period of up to 5 years are represented by the estimates (with 95% confidence intervals) predicted by the model. *p* values were considered significant at ≤0.05. Since the LME model does not account for changes in ASMs and seizure frequency during VNS therapy, we performed an additional descriptive analysis to demonstrate the changes in relevant clinical features at the individual patient level.

## 3 Results

### 3.1 Changes in TMT-A, TMT-B and DB z scores during follow-up

**Figure 1:**
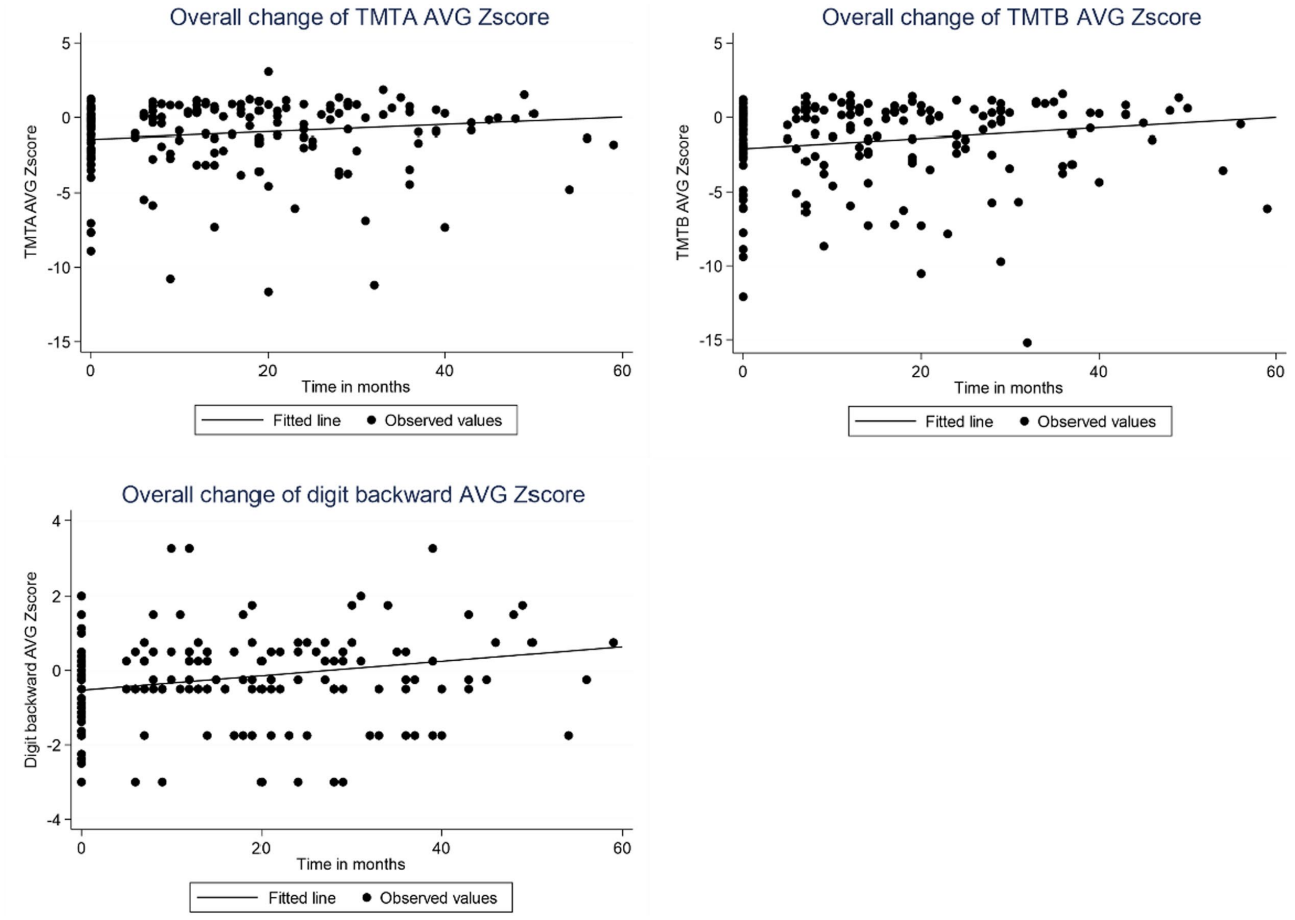
Observed TMT-A, TMT-B and DB z scores and fitted curve based on linear mixed-effects model for all patients over time following VNS therapy. The baseline z scores for TMT-A, TMT-B and DB were − 1.42, −2.01, and − 0.53, respectively. AVG = Average.

Baseline z scores for TMT-A, TMT-B and DB were − 1.42, −2.01 and − 0.53, respectively. According to the LME model, significant improvements were observed in TMT-A, TMT-B, and DB z scores following VNS therapy during a follow-up duration of up to 5 years. On average, TMT-A z scores improved by 0.024 units (*p* = 0.009), TMT-B z scores by 0.034 units (*p* = 0.003), and DB z scores by 0.019 units per month (*p* < 0.001) (Table 2 and Figure 1). These changes correspond to improvements of 0.58 SD at 2 years and 1.44 SD at 5 years for TMT-A, 0.82 SD at 2 years and 2.04 SD at 5 years for TMT-B, and 0.46 SD at 2 years and 1.14 SD at 5 years for DB.

**Table 2:** Number of patients who improved clinically significantly in different baseline performance categories and average z score change per month in TMT-A, TMT-B and DB z scores depending on the baseline performance category.

Tests	Patients with clinically significant improvement	Changes in Z scores (units/month)			
	N (%)	Average change	95% CI	*p-value*	Number of patients and (tests)
**TMT-A**					
All patients#	22/45 (49%)	**0.024**	0.006–0.042	**0.009**	45 (141)
*Normal*	5/22 (23%)	**0.012**	0.004–0.019	**0.003**	22 (63)
*Mild impairment*	4/5 (80%)	0.004	−0.066–0.058	*0.906*	5 (14)
*Moderate impairment*	8/10 (80%)	0.045	−0.007–0.096	*0.094*	10 (36)
*Severe impairment*	5/8 (63%)	0.018	−0.032–0.067	*0.478*	8 (28)
**TMT-B**					
All patients#	24/45 (53%)	**0.034**	0.012–0.057	**0.003**	45 (141)
*Normal*	8/21 (38%)	**0.012**	0.001–0.023	**0.032**	21 (65)
*Mild impairment*	6/8 (75%)	**0.055**	0.018–0.091	**0.003**	8 (24)
*Moderate impairment*	2/5 (40%)	−0.007	−0.07–0.056	*0.821*	5 (16)
*Severe impairment*	8/11 (80%)	0.067	−0.004–0.139	*0.067*	11 (36)
**DB**					
All patients#	18/45 (40%)	**0.019**	0.011–0.028	**<0.001**	45 (137)
*Normal*	4/24 (17%)	0.006	−0.006–0.017	*0.357*	24 (81)
*Mild impairment*	10/16 (63%)	0.008	0.009–0.024	*0.365*	16 (46)
*Moderate impairment*	3/4 (75%)	0.029	−0.025–0.08	*0.292*	4 (10)
*Severe impairment*	1/1 (100%)	^*^			

#Each test, TMT-A, TMT-B and DB, had one missing result for the baseline z-score (across different patients), resulting in a total of 45 patients. ^*^Analysis could not be performed for this category due to only one patient. In ‘average change’ and ‘*p*-value’ columns, bold values denote ‘significant change’ and ‘statistically significant *p*-values’, respectively.

**Figure 2:**
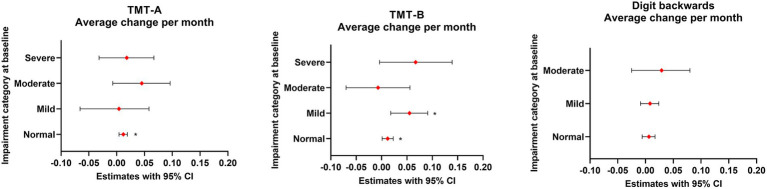
Average change in TMT-A, TMT-B and DB z scores during the follow-up period in different baseline performance categories. ^*^Statistically significant.

The highest proportion of patients achieved clinically significant improvement in TMT-B, followed by TMT-A and DB (53, 49 and 40%, respectively) (Table 2 and Figure 2). Furthermore, when the baseline performance was normal, a noticeably higher percentage of patients demonstrated clinically significant improvements in TMT-B than in TMT-A or DB (38, 23 and 17%, respectively), although average monthly improvement was equal both in TMT-A and TMT-B z scores (0.012 units/month, *p* = 0.003 and *p* = 0.032, respectively). The number of patients with severely impaired performance at baseline was higher in TMT-B compared to TMT-A and DB. However, 80% of the patients with severely impaired performance in the TMT-B at baseline achieved clinically significant improvement, and the average z score improvement was 0.067 units per month (*p* = 0.067), which was the most prominent among all groups.

Individual changes in TMT-A, TMT-B and DB z scores as well as changes in ASM use and seizure frequency during the follow-up period are presented in Supplementary Tables S1–S3.

### 3.2 Effect of psychiatric comorbidities on TMT-A, TMT-B, and DB performance

At baseline, patients with psychiatric comorbidities (ten had depression, one had bipolar disorder, and one had psychosis) had on average 1.62 units lower TMT-A (*p* = 0.112), 2.03 units lower TMT-B (*p* = 0.079), and 0.79 units lower DB z scores (*p* = 0.026) compared to patients without psychiatric comorbidities (Table 3).

**Table 3:** Baseline values and changes in z-scores per month for overall patients as well as in different clinical categories.

Clinical parameters	N (%)	TMT-A Z score				TMT-B Z score				DB Z score			
		Baseline	Change	95% CI	*p-value*	Baseline	Change	95% CI	*p-value*	Baseline	Change	95% CI	*p-value*
All patients	46 (100)	−1.42	0.024	0.006–0.042	**0.009**	−2.01	0.034	0.012–0.057	**0.003**	−0.53	0.019	0.011–0.028	**<0.001**
Psychiatric comorbidity													
*No*	34 (73.9)	−0.99	0.018	−0.01–0.040	*0.13*	−1.55	0.025	0.004–0.047	**0.018**	−0.34	0.017	0.007–0.026	**0.001**
*Yes*	12 (26.1)	−2.61	0.042	0.014–0.07	**0.003**	−3.58	0.058	0.004–0.113	**0.036**	−1.13	0.028	0.009–0.046	**0.003**
Epilepsy type													
*FLE*	18 (39.1)	−1.57	0.030	−0.01–0.07	*0.142*	−2.38	0.032	−0.014–0.079	*0.172*	−0.80	0.013	0.0–0.026	0.055*
*TLE*	17 (37)	−1.79	0.024	0.002–0.044	**0.028**	−2.37	0.040	0.006–0.074	**0.020**	−0.74	0.012	−0.003–0.026	*0.120*
*Other*	11 (23.9)	−0.89	−0.009	−0.084–0.065	*0.810*	−1.13	0.008	−0.036–0.053	*0.717*	0.18	0.003	−0.018–0.023	*0.795*
Predominant seizure type													
*FAS/FIAS*	34 (73.9)	−1.17	0.019	−0.005–0.043	*0.123*	−1.74	0.034	0.009–0.058	**0.006**	−0.45	0.018	0.008–0.029	**0.001**
*FBTCS*	12 (26.1)	−2.37	0.035	0.009–0.061	**0.008**	−3.12	0.025	−0.029–0079	*0.37*	−0.73	0.018	0.004–0.033	**0.013**
ASMs													
*1–2*	17 (37)	−0.90	0.042	0.015–0.068	**0.002**	−0.98	0.042	0.014–0.071	**0.004**	−0.10	0.014	−0.003–0.031	*0.114*
*3–4*	29 (63)	−1.77	0.012	−0.011–0.035	*0.312*	−2.76	0.030	−0.001–0.060	*0.058*	−0.80	0.023	0.013–0.033	**<0.001**

ASM, antiseizure medication; FAS, focal aware seizure; FBTCS, focal to tonic clonic seizure; FIAS, focal impaired awareness seizure; FLE, frontal lobe epilepsy; TLE, temporal lobe epilepsy. Bold values indicate statistically significant *p*-values.

During the follow-up period, TMT-A, TMT-B and DB z scores improved significantly for patients with psychiatric comorbidities (0.042 units/month, *p* = 0.003; 0.058 units/month, *p* = 0.036; and 0.0028 units/month, *p* = 0.003, respectively) (Table 3 and Supplementary Figure S2). These changes correspond to improvements of 1.0 SD at 2 years and 2.5 SD at 5 years in TMT-A, 1.4 SD at 2 years and 3.5 SD at 5 years in TMT-B, and 0.67 SD at 2 years and 1.7 SD at 5 years in DB. The improvement in TMT-A and TMT-B was more than twofold for patients with psychiatric comorbidities compared to patients without psychiatric comorbidities; in DB, the increase was similar for both groups. Psychiatric comorbidity subgroups showed clinically significant improvements in TMT-A (58% with, 45% without), TMT-B (67% with, 48% without) and DB (67% with, 30% without), accordingly (Supplementary Tables S1–S3).

### 3.3 Effect of epilepsy and predominant seizure type on TMT-A, TMT-B and DB performance

The baseline z scores for different epilepsy and predominant seizure types are presented in Table 3. During the follow-up period, improvements in TMT-A, TMT-B and DB were similar in FLE and TLE patients, whereas patients with other types of epilepsy exhibited only slight improvements in TMT-B and DB z scores, and TMT-A z scores even decreased (Supplementary Figure S3). Furthermore, the improvement in TMT-A z scores was almost twofold for patients with FBTCS compared to patients with FAS/FIAS (*p* = 0.38), whereas TMT-B and DB z scores improved similarly for FBTCS and FAS/FIAS patients (Table 3 and Supplementary Figure S4). Correspondingly, more patients with FBTCS improved clinically in TMT-A and DB, whereas in TMT-B, the percentage of clinically significantly improved patients was almost identical between the two seizure groups (Supplementary Tables S1–S3).

Importantly, 57% of the seizure responders (≥ 50% reduction) exhibited clinically significant improvement in TMT-A, 62% in TMT-B, and 33% in DB during the follow-up period. In comparison, among the cognitive responders, 45.5% in TMT-A, 46% in TMT-B and 61% in DB were non responders for their predominant seizure type (Supplementary Tables S1–S3).

### 3.4 Effect of ASMs on TMT-A, TMT-B, and DB performance

Baseline z scores for patients taking 1–2 ASMs and 3–4 ASMs are presented in Table 3. During the follow-up period, TMT-A and TMT-B z scores improved significantly for patients taking 1–2 ASMs (0.042 units/mo, *p* = 0.002; 0.042 units/mo, *p* = 0.004, respectively), and DB z scores improved significantly for patients taking 3–4 ASMs (0.023 units/mo, *p* < 0.001) (Table 3 and Supplementary Figure S5). In TMT-A, the increase was over threefold for patients taking 1–2 ASMs compared to patients taking 3–4 ASMs. Additionally, approximately half of the patients who had a reduced ASM burden during follow-up experienced clinically significant improvements in all three tests. Conversely, among those patients who did not undergo a reduction in AMS burden during follow-up, 40% showed a clinically significant improvement in TMT-A, 56% in TMT-B, and only 28% in DB (Supplementary Tables S1–S3).

## 4 Discussion

The purpose of our study was to investigate the variability in three cognitive tests assessing differential aspects of executive functions and working memory in a group of DRE patients receiving VNS therapy during a follow-up duration of up to 5 years. The key finding was that performance in all tests improved during the follow-up period at the group level, with the most robust improvement observed in TMT-B, which requires inhibition control and set-switching in addition to the visuoperceptual processing speed needed in TMT-A and working-memory performance essential in DB. Moreover, the improvement in TMT-B was further enhanced if the baseline performance was impaired and the patient had psychiatric comorbidities.

The predicted improvement in TMT-B z scores was 0.034 units per month, which was noticeably higher than that in TMT-A and almost twofold the change observed in DB z scores. Similarly, in descriptive analysis, more patients improved in TMT-B than in the two other tests. This robust improvement observed in TMT-B performance is unlikely to be explained by practice effect, as previous studies have indicated that TMT-A is more susceptible to practice effects than TMT-B (20, 31, 32). Although the possibility of achieving clinically significant improvement was higher in all the tests if the baseline performance was impaired, in the TMT-B, a large proportion of patients with normal baseline performance also experienced similar improvements. The TMT-B is supposed to assess higher components of executive functions than the TMT-A or DB test. Accordingly, the more robust improvement observed in TMT-B performance in comparison to TMT-A and DB could indicate that higher cognitive functions such as inhibition control and set-switching, which are more specifically involved in TMT-B performance, may be enhanced even more by VNS than, for example, psychomotor speed. Interestingly, in a study in which DRE patients treated with neurostimulation were evaluated with comprehensive neuropsychological examination, among the individual cognitive tests, performance was most severely impaired in the TMT, particularly in Part B (33).

In all three cognitive tests, baseline performance had a clear effect on the probability of experiencing clinically significant improvement during follow-up, as there were more cognitive responders in patients with impaired performance than in patients with normal performance. This is consistent with our preceding study in which we investigated changes in AEF during VNS therapy using repeated EpiTrack evaluations and observed that a markedly higher percentage of the patients demonstrated clinically meaningful improvement during follow-up if the baseline performance was impaired (18). However, in the present study, the possibility of experiencing clinically significant improvement with normal baseline performance was distinctly higher in TMT-B than in the other two tests. Accordingly, patients with good cognitive functioning at baseline can also benefit cognitively from VNS therapy.

When evaluating different clinical variables and performance in these three tests, we observed that the robust improvement in TMT-B in comparison to TMT-A and DB was even more pronounced in patients with psychiatric comorbidities. Furthermore, patients with psychiatric comorbidities had over twofold higher increases in TMT-A and TMT-B z scores compared to patients without psychiatric comorbidities during follow-up, whereas in DB z scores the change was almost identical. These results support our previous findings (18) on the dynamic effect of psychiatric comorbidities on executive functions as well as the potential of VNS, probably via improved mood, to enhance executive function performance in patients with DRE. The enhancement of executive functions during VNS therapy appears to focus specifically on higher cognitive functions in patients with psychiatric comorbidities.

Epilepsy type is one of the main static factors influencing the cognitive functioning of epilepsy patients (34). In our study, patients with TLE and FLE had similar improvements in the three tests, and these changes were also concordant with the whole study group, which is in contrast with some previous studies reporting differences in cognitive test results between TLE and FLE patients (35). Conversely, patients with other types of epilepsy did not improve during follow-up.

High seizure frequency and intensity are among the seizure-related factors that are usually associated with cognitive impairment in patients with epilepsy (36). During the follow-up period, changes in the z scores of the three tests were otherwise similar for FAS/FIAS and FBTCS patients and consistent with the overall study population; however, patients with FBTCS experienced the greatest improvement in TMT-A, and that improvement was almost twofold compared to the improvement in the FAS/FIAS group. In individual analysis, the correlation between seizure responders and clinically significant improvement was more evident in TMT-A/B than in DB. In previous studies, a longer duration of active epilepsy has been associated with worse TMT-B performance (19), and in a follow-up study, only patients with improved seizure control due to ASM changes also improved in TMT-B but not in TMT-A (20). However, in our study, the decrease in seizure frequency was not always concordant with clinically significant improvement in any of the tests highlighting the involvement of other factors in addition to seizure reduction.

In general, it is believed that the higher the number of concomitant ASMs, the worse the cognitive performance is (12, 18, 37). In our study, patients treated with 1–2 ASMs experienced greater increases in TMT-A and TMT-B z scores during the follow-up period compared to patients taking 3–4 ASMs, and for TMT-A, the improvement was more than threefold. These findings are similar to the results in our previous study, where patients taking 1–2 ASMs exhibited almost quadruple improvement in the EpiTrack total score compared to patients taking 3–4 ASMs (18). In contrast, DB z scores increased significantly for patients taking 3–4 ASMs, which could indicate that a high number of concomitant ASMs does not affect working memory performance as much as it affects other components of executive functions. Moreover, in a recent study of 132 focal epilepsy patients evaluated with TMT-A and TMT-B, ASM polypharmacy was associated with worse performance in TMT-A (20). However, in individual analysis, firstly, a decrease in ASM burden was not always concordant with improvement in z scores. Secondly, more than half of the patients without AMS reduction experienced clinically significant improvement in TMT-B, supporting an additive direct effect of VNS on AEF.

Neuroimaging studies have indicated that large-scale brain networks, including prefrontal and parietal structures, mediate TMT performance (25). The frontal lobe, especially the prefrontal cortex, has been associated with the TMT due to its role in executive functions, such as attention and planning (38–41), whereas the temporal lobe may be engaged in working memory demands of recalling numbers and letters during the TMT-B (38, 40). Moreover, the occipital and parietal lobes have been linked to TMT performance due to their involvement in visual search abilities (41). fMRI studies have demonstrated that TMT-B performance is mediated by the same brain networks as TMT-A performance with some additional areas, particularly the left dorsolateral prefrontal cortex and inferior frontal gyrus (41). Activation of these brain regions during TMT-B performance is consistent with increased sensorimotor and visual–spatial processing demands required for performing TMT-B compared to TMT-A (38, 39). The right IFG is engaged during set-switching (39), which is essential in performing TMT-B successfully (22). Performance in DB has been associated with greater frontal activation than performance in the Digit Span Forward test in neuroimaging studies conducted on healthy adults (42). Furthermore, manipulation of information during working memory tests, such as DB, also requires activation of posterior brain regions (e.g., superior and inferior parietal cortex, superior temporal cortex), indicating a role for nonfrontal brain regions (42, 43).

Previously, the immediate effects of VNS on human working memory performance have been linked to increased brain levels of noradrenaline due to activation of the locus coeruleus (13). The differential effects on VAN-related networks and brain regions by stimulation of the vagal nerve in fMRI studies are complex, including responses within insular, frontal, temporal, and occipital cortices (44, 45), and require further studies addressing activation of distinct brain networks in relation to specific cognitive tests during VNS.

Our current results support the importance of evaluating individual cognitive tests separately in addition to a more global assessment of AEF and other cognitive domains, since the performance in each test is distinct both at baseline and during active VNS therapy. By assessing the performance of individual tests as part of a more comprehensive cognitive evaluation, we can better identify cognitive profiles of patients with DRE who are more likely to benefit cognitively from VNS therapy. Our results suggest that TMT-B is the most sensitive test for VNS response. Consequently, patients with psychiatric comorbidities and poor performance on TMT-B at baseline could be expected to gain significant cognitive benefits from VNS therapy.

## 5 Strengths and limitations

The main limitation of our study is the retrospective uncontrolled design and analysis of the data collected according to the clinical protocol. Due to the COVID-19 pandemic, the scheduled visits did not always take place according to our clinical VNS follow-up protocol, with a mean follow-up duration of 31.5 months. Therefore, changes in the z scores over time were analyzed using a statistical model to compensate for the variation in time points and the numbers of tests of individual patients when predicting result changes per month during a period up to 5 years. In addition, the LME model did not take into account possible modifications to ASMs, seizure status changes or variations in the severity of depression during the follow-up period. On the other hand, the use of the LME model yielded a statistically robust evaluation of the z scores as time series data after VNS implantation. Finally, practice effects on neuropsychological tests may have contributed to improvements in the retests.

## 6 Conclusion

A gradual improvement in all three VAN-related cognitive tests was observed after the initiation of VNS therapy. The improvement in TMT-B performance during the follow-up period was superior to that in TMT-A or DB. This could indicate that higher cognitive functions, such as inhibition control and set-switching, may be enhanced even more by VNS than psychomotor speed or working memory.

## Data availability statement

The original contributions presented in the study are included in the article/Supplementary materials, further inquiries can be directed to the corresponding author.

## Ethics statement

This was a non-interventional study in which data was collected prospectively but analyzed retrospectively from a VNS quality register at Tampere University Hospital, therefore not requiring ethics committee approval according to Finnish Law on Research.

## Author contributions

NL: Conceptualization, Writing – original draft, Writing – review & editing. PB: Data curation, Formal analysis, Methodology, Supervision, Writing – review & editing. JR: Data curation, Formal analysis, Methodology, Software, Writing – review & editing. LK: Investigation, Writing – review & editing. KL: Conceptualization, Supervision, Writing – review & editing. ER-O: Data curation, Methodology, Resources, Writing – review & editing. JP: Conceptualization, Investigation, Project administration, Supervision, Validation, Writing – review & editing.

## Figures and Tables

**Table:** 

AEF	attention and executive function
ASM	antiseizure medication
DB	Digit Span Backward
DMN	default mode network
DRE	drug-resistant epilepsy
GGE	genetic generalized epilepsy
FAS	focal aware seizure
FBTCS	focal to bilateral tonic–clonic seizure
FIAS	focal impaired awareness seizure
FLE	frontal lobe epilepsy
fMRI	functional magnetic resonance imaging
LME	linear mixed-effects
TLE	temporal lobe epilepsy
TMT	Trail-Making Test
VAN	vagus afferent network
VNS	vagus nerve stimulation
