# Supplementary material for: Complex executive functions assessed by the trail making test (TMT) part B improve more than those assessed by the TMT part A or digit span backward task during vagus nerve stimulation in patients with drug-resistant epilepsy
Source: Front Psychiatry. 2024 Feb 14;15:1349201. doi: 10.3389/fpsyt.2024.1349201 (PMC10899669; doi:10.3389/fpsyt.2024.1349201)
Supplement: Supplementary file 1 [file Data_Sheet_1.PDF]

Supplementary Material

Complex executive functions assessed by the Trail Making Test (TMT) Part B improve more than those assessed by the TMT Part A or Digit Span Backward task during vagus nerve stimulation in patients with drug-resistant epilepsy

Niina Lähde[1,2*], Pabitra Basnyat[2], Jani Raitanen[3], Leena Kämppi[4], Kai Lehtimäki[5], Eija Rosti-Otajärvi[1,6], Jukka Peltola[1,2]

1. Department of Neurology, Tampere University Hospital, Tampere, Finland

2. Faculty of Medicine and Health Technology, Tampere University, Tampere, Finland

3. Faculty of Social Sciences, Health Sciences, Tampere University, Tampere, Finland; UKK Institute for Health Promotion Research, Tampere, Finland

4. Epilepsia Helsinki, Member of EpiCARE ERN, Department of Neurology, Helsinki University Hospital and University of Helsinki, Helsinki, Finland

5. Department of Neurosurgery, Tampere University Hospital, Tampere, Finland

6. Department of Rehabilitation and Psychosocial Support, Tampere University Hospital, Tampere, Finland

Corresponding author's details

Niina Lähde, MD

Tampere University Hospital

Department of Neurology

Biokatu 8, 33520 Tampere, Finland

niina.j.lahde@tuni.fi, nlahde@gmail.com

Tel: +358 452 392 308

*** Correspondence:** niina.j.lahde@tuni.fi

Supplementary results

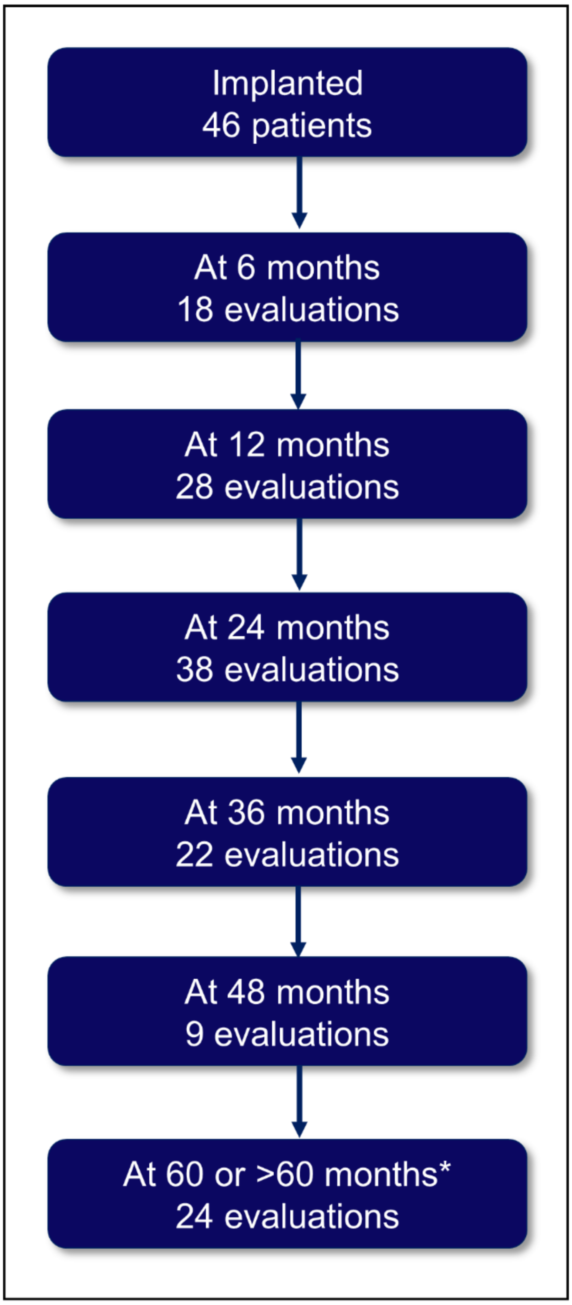

Supplementary fig 1. The flowchart of the follow-up protocol. *Summary of the cognitive evaluations at different time points after VNS implantation. Each time point represents a specific duration in months as follows: 6 months (ranging from 4 to 8 months), 12 months (ranging from 8 to 18 months), 24 months (ranging from 18 to 30 months), 36 months (ranging from 30 to 42 months), 48 months (ranging from 42 to 54 months), 60 months (54 to 66 months). *5 evaluations at 60 months and 19 evaluations between 72 to 108 months.*

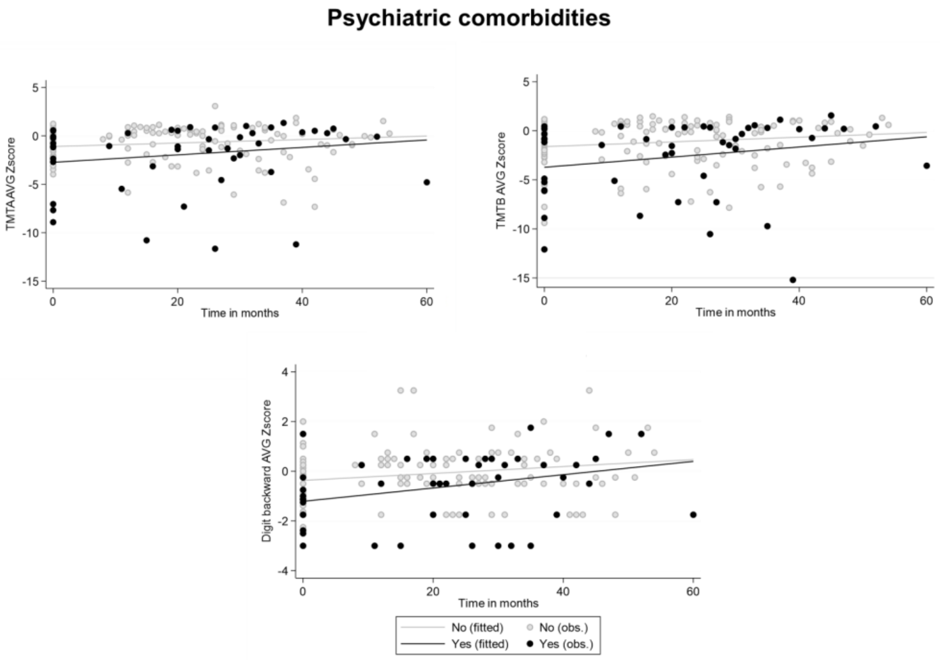

Supplementary figure 2*. Observed TMT-A, TMT-B and DB z scores, and fitted curve based on linear mixed-effects model over time following VNS therapy based on presence of psychiatric comorbidities*.

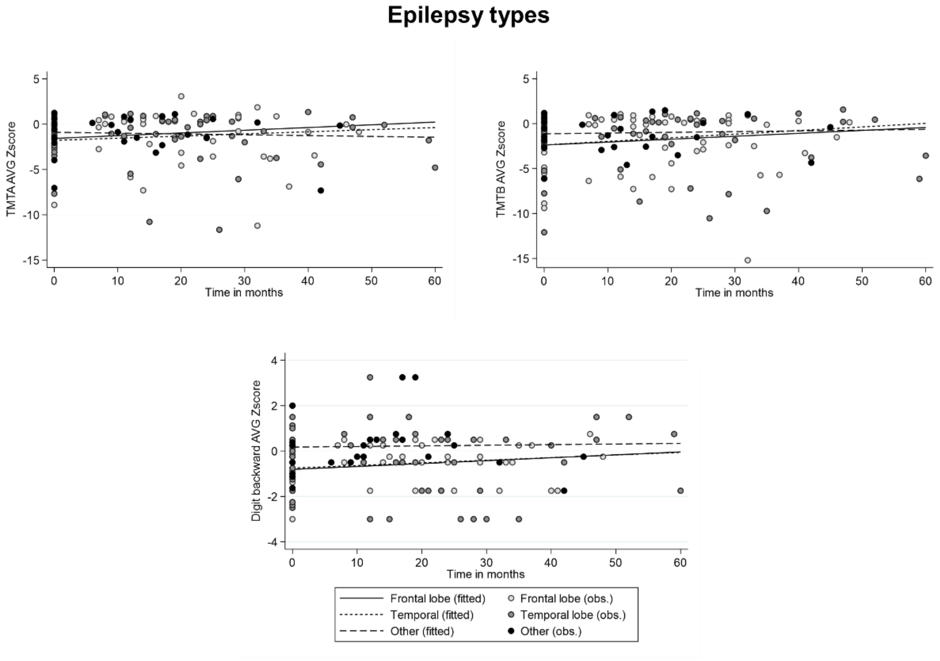

Supplementary figure 3. *Observed TMT-A, TMT-B, and DB z scores and fitted curve based on linear mixed-effects model over time following VNS therapy based on epilepsy type.*

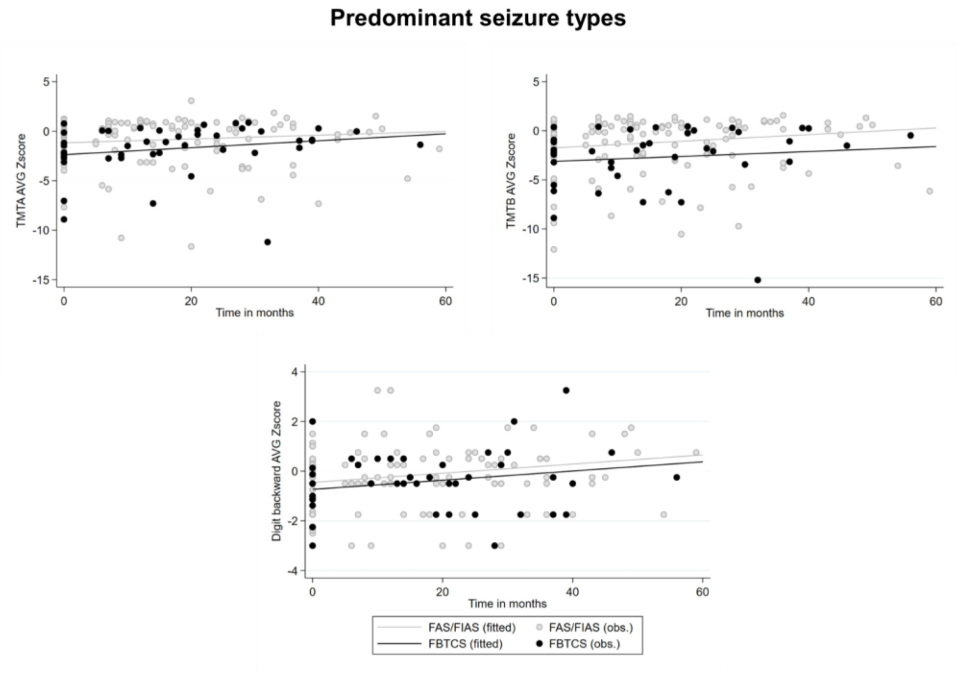

Supplementary figure 4. *Observed TMT-A, TMT-B and DB z scores and fitted curve based on linear mixed-effects model over time following VNS therapy based on predominant seizure types.*

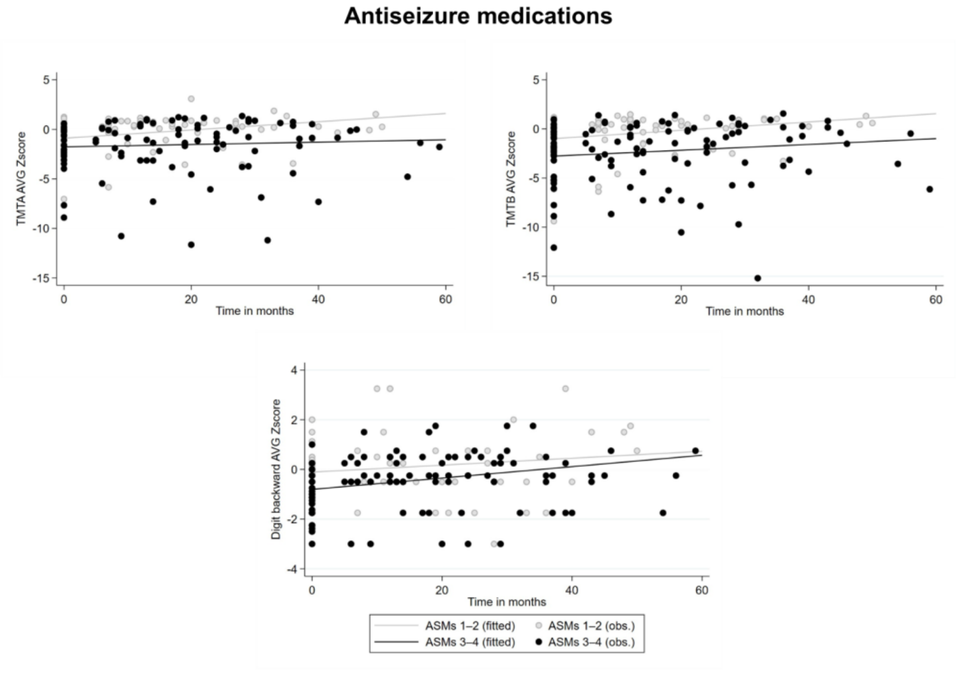

Supplementary figure 5. *Observed TMT-A, TMT-B and DB z scores and fitted curve based on linear mixed-effects model over time following VNS therapy based on the number of ASMs in use.*

Supplementary table 1. *Individual changes on ASMs and predominant seizure type during follow-up in patients with FLE*

Age/sex	Psychiatric comorbidities	ASMs at baseline	ASMs at final observation point	Predominant seizure type	Monthly seizure frequency 12 months prior to baseline	Change in predominant seizure type compared to baseline	Duration of VNS therapy (months)	Change in TMTA Z score	Change in TMTB Z score	Change in DB Z score
**M/24**	No	OXC 1800, PER 12, CLB 90	OXC 1800, PER 12, CLB 80↓	FAS	30	↓ < 25%	31	-2.03 → -6.88	-5.21 → -5.69	-1.75 → 0.25
**M/34**	Yes/present	LTG 400, PGB 525, CLB 20, VPA 1500	LTG 400, PGB 225↓, CLB 20, VPA %	FAS	Daily	↓ 100%	71	-0.22 → 1.53	-4.88 → 1.19	-1.75 → 0.5
**F/41**	No	LCM 600	LCM 600	FIAS	17	↓ ≥ 50% < 75%	31	-2.73 → 1.86	-1.99 → 0.92	-1.75 → -1.75
**F/28**	No	OXC 1200, ZNS 400	OXC 1200, ZNS 250 ↓	FIAS	2	↓ ≥ 50% < 75%	29	-7.70 ↑ -3.45	-9.40 ↑ -3.27	-1.75 → -1.75
**F/33**	No	LTG 500, TPM 400	LTG 500, TPM 50 ↓	FIAS	0,3	↓ 100%	14	-0.47 ↑ 0.53	-0.17 ↑ 0.92	-0.25 → 0
**M/32**	No	LEV 2000, ESL 2000	BRV 200, ESL 2000	FIAS	3	↓ ≥ 75% < 90%	29	-2.64 → 1.02	-0.11 ↑ 0.92	-0.88 → -1.25
**M/57**	No	CLB 20, CBZ 1100	CLB 20, LTG 100, LCM 600 +,	FIAS	200	↑ < 25%	76	0.20 → 1.35	0.28 → 1.08	-1.75 → -1.0
**M/39**	Yes/past	LCM 350, CBZ 1100	LCM 400↑, CBZ 1100	FIAS	7	↓ ≥ 50% < 75%	20	-0.08 → 0.86	-0.83 ↑ 0.34	-0.25 → -0.5

**F/28**	No	LCM 600, BRV 200, PER 2	LCM 600, BRV 200, PER 4 ↑	FIAS	0,5	↓ 100%	12	-0.33 → 0.44	0.2 → 1.05	0 → 0.25
**M/46**	Yes/present	LCM 400, CLB 30, CBZ 1000	LCM 600, CLB 30, CBZ %	FIAS	17	↓ ≥ 50% < 75%	75	0.58 → 0.73	0.45 → 0.11	-1 → 0.75
**F/70**	No	LTG 400, PER 10, CLB 30	LTG 400, PER 8 ↓, CLB %	FIAS	10	↓ < 25%	42	-1.62 → -0.85	-2.36 → 0.15	-0.25 → -0.25
**M/29**	No	OXC 2100, ZNS 500, CLB 20	OXC 2100, ZNS 50 ↓, CLB 20	FBTCS	SF	No change	29	0.25 → 0.77	0.07 → 0.41	-0.88 → 0
**F/32**	No	LEV 3000, LCM 600	LEV 3000, LCM 600, CLB 5 +	FBTCS	0,3	↓ < 25%	29	0.76 → 0.87	0.38 → -0.12	0 → 0.25
**M/29**	No	LCM 600, PER 8, CLB 20	LCM 600, PER 8, CLB 20	FBTCS	0,3	↓ 100%	12	-0.14 → 0.32	-1.76 → 0.14	-0.88 → 0
**F/23**	No	LEV 3000, LCM 500	LEV 3000, LCM 500	FBTCS	1	↓ 100%	25	-2.16 → -1.87	-2.41 → -2.08	-0.13 → -1.75
**M/46**	No	LTG 300, VPA 1200, TPM 400	LTG 300, VPA 1200, TPM 400	FBTCS	1	↓ ≥ 50% < 75%	46	-2.71→ -0.02	-5.53 → -1.51	-1.38 → 0.75
**F/33**	Yes/past	ZNS 500, ESL 1600, CLB 30, LEV 1500	ZNS 500, ESL 1600, CLB % LEV %	FBTCS	1	↑ 100%	32	-8.92 → -11.2	-8.88 → -15.2	-3 → -1.75
**F/45**	No	LTG 400, PER 8, LEV 2000, TPM 400	LTG 400, PER 8, LEV %, TPM %	FBTCS	1	↓ 100%	37	-3.12 → -1.68	-3.20 → -3.15	-1 → -0.25

↓= decrease/reduction, ↑ = increase, + = add on, % = withdrawn. ASM, antiseizure medication; FAS, focal aware seizure; FBTCS, focal to bilateral tonic clonic seizure; FIAS, focal impaired awareness seizure; FLE, frontal lobe epilepsy; TLE, temporal lobe epilepsy; BRV, brivaracetam; CBZ, carbamazepine; CLB, clobazam; ESL, eslicarbazepine acetate; LEV, levetiracetam; LCM, lacosamide; LTG, lamotrigine; OXC, oxcarbamazepine; PGB, pregabalin; PER, perampanel; TPM, topiramate; VPA, valproate; ZNS, zonisamide; LOCF, last observation carried forward.. The green boxes in the 'ASMs at LOCF' column

highlights ASM decrease/reduction, while the red boxes indicate ASM burden increase. In the 'Change in predominant seizure type from baseline' column, green highlights more than 50% seizure reduction, and red indicates more than 50% seizure increase. In TMT-A, TMT-B and DB change' column, green highlights patients with clinically significant improvement, while red signifies those with worsening.

Supplementary table 2. *Individual changes on ASMs and predominant seizure type during follow-up in patients with TLE.*

Age/sex	Psychiatric comorbidities	ASMs at baseline	ASMs at final observation point	Predominant seizure type	Monthly seizure frequency 12 months prior to baseline	Change in predominant seizure type compared to baseline	Duration of VNS therapy (months)	Change in TMTA Z score	Change in TMTB Z score	Change in DB Z score
**F/44**	No	BRV 200, LCM 400	BRV 200, LCM 400	FAS	54	↓ < 25%	24	-0.06 → 0.89	0.56 → 1.14	1.13 → 0.75
**M/33**	Yes/present	ZNS 300, OXC 1800, PGB 225	ZNS 200 ↓, OXC 1800, PGB %	FAS	70	↓ ≥ 75% < 90%	35	0.53 → 0.75	-0.32 → 1.57	-2.38 → 0.5
**M/22**	No	BRV 200, ESL 1600	BRV 200, ESL 1600	FIAS	12	↓ 100%	26	0.66 → 0.27	-0.48 → 0.46	0.5 → 0.5
**F/31**	Yes/past	LCM 400, CLB 50	LCM 400, CLB 35↓, BRV 150 +	FIAS	8	↓ ≥ 25 < 50%	48	-0.78 → -0.08	0.23 → 0.45	1.5 → 1.5
**F/24**	No	LTG 500, LEV 1750, ZNS 200	LTG 500, LEV 1000↓ ZNS ٪	FIAS	1	↑ 100%	33	-0.06 → 0.44	-0.27 → -0.12	1 → 0.5
**M/40**	No	LCM 200, LTG 400, LEV 1000	LCM 200, LTG 400, BRV 100	FIAS	5	↓ ≥ 25% < 50%	34	0.16 → 0.64	0.98 → 0.92	0 → 1.75
**F/33**	No	LCM 600, PGB 600, CLB 40	LCM 600, PGB 600, CLB 25↓	FIAS	5	↓ < 25%	18	-0.22 → 1.22	0.92 → -0.24	0 → 1.5

**F/19**	Yes/present	OXC 2100, ZNS 400, LEV 1000	OXC 2100, ZNS 200 ↓, LEV %	FIAS	5	↑ ≥ 25 % < 50 %	29	-2.69 → 0.66	-0.83 → -0.58	-0.75 → 0.5
**F/22**	No	LCM 500, BRV 150, ZNS 200	LCM 500, BRV 200 ↑, ZNS %	FIAS	2	↓ ≥ 75% < 90%	18	-0.65 → 0.00	-1.75 → 0.56	-1.13 → -1.75
**F/41**	No	LTG 200, ZNS 300, VPA 600	LTG 200, ZNS 300, VPA 600, CLB 10 +	FIAS	1	↓ 100%	60	-3.48 → -0.65	-2.74 → -4.98	-0.25 → -1.75
**M/32**	No	LTG 400, ZNS 400, CLB 40	LTG 400, ZNS 400, CLB 20↓	FIAS	4	↓ ≥ 25% < 50%	35	-3.08 → -4.44	-7.76 ↑ -3.76	-1.13 → -0.5
**F/45**	Yes/past	LCM 400, TPM 400, BRV 200	LCM 400, TPM 100 ↓ BRV 200	FIAS	5	↓ ≥ 25% < 50%	24	-2.35 → -1.99	-5.25 → -1.83	-2.5 → -3
**M/32**	Yes/present	LTG 400, ZNS 500, CLB 20	LTG 400, ZNS %, CLB %,	FIAS	Daily	↓ 100%	53	-7.68 ↑ -4.79	-12.09 ↑ -3.56	-1.75 → -1.75
**F/31**	Yes/ present	LCM 200, LTG 500, ZNS 400	LCM 400↑, LTG 550 ↑, BRV 100	FIAS	NA	NA	95	-1.09 → 0.87	-6.07 ↑ -1.90	-1.25 → 0.5
**F/32**	Yes/ present	LTG 400, TPM 450	LTG 400, TPM 325 ↓	FBTCS	NA	NA	40	-1.18 → 0.28	-1.15 → 0.25	-1.13 → -0.5
**M/43**	No	LCM 600, ZNS 300, CLB 10	LCM 600, ZNS 150 ↓, CLB 15 ↑	FBTCS	1	↓ 100%	56	-2.23 → 0.58	-1.94 → -0.75	-2.25 → -1.75
**M/28**	No	LEV 2500, OXC 1200, LCM 400	BRV 175, PER 8, LCM 600 ↑	FBTCS	1	↓ < 25%	83	-2.51 → 0.17	-2.19 → -0.55	-2.25 → -0.5

↓= decrease/reduction, ↑ = increase, + = add on, % = withdrawn. ASM, antiseizure medication; FAS, focal aware seizure; FBTCS, focal to bilateral tonic clonic seizure; FIAS, focal impaired awareness seizure; FLE, frontal lobe epilepsy; TLE, temporal lobe epilepsy; BRV, brivaracetam; CLB, clobazam; ESL, eslicarbazepine acetate; LEV, levetiracetam; LCM, lacosamide; LTG, lamotrigine; OXC, oxcarbamazepine; PGB, pregabalin; PER, perampanel; TPM, topiramate; VPA, valproate; ZNS, zonisamide; LOCF, last observation carried forward.. The green boxes in the 'ASMs at LOCF' column highlights ASM decrease/reduction, while the red boxes indicate ASM burden increase. In the 'Change in predominant seizure type from baseline' column, green highlights

more than 50% seizure reduction, and red indicates more than 50% seizure increase. In TMT-A, TMT-B and DB change' column, green highlights patients with clinically significant improvement, while red signifies those with worsening.

Supplementary table 3. *Individual changes on ASMs and predominant seizure type during follow-up in patients with other types of epilepsy.*

Age/sex	Psychiatric comorbidities	ASMs at baseline	ASMs at final observation point	Predominant seizure type	Monthly seizure frequency 12 months prior to baseline	Change in predominant seizure type compared to baseline	Duration of VNS therapy (months)	Change in TMTA Z score	Change in TMTB Z score	Change in DB Z score
**M/51**	No	LEV 1000, ESL 1200, PER 6	LEV 1000, ESL 1200, PER %	FAS	10	↑ > 100%	46	-2.07 → -3.14	-1.47 → -2.56	0.25 → -0.25
**M/53**	No	LCM 400, LEV 2000	LCM 400, LEV 2000	FIAS	10	↓ < 25%	13	1.26 → 1.11	1.19 → 1.48	2 ↑ 3.25
**M/36**	No	LEV 2500, OXC 1200	BRV 200, OXC 1200	FIAS	4	↓ ≥ 75% < 90%	92	1.1 → 1.42	1.18 → 1.15	-1 → 0.5
**F/26**	No	LEV 3000, LCM 500	LEV 3000, LCM 500	FIAS	0,5	↓ < 25%	32	0.64 → 0.19	0.65 → 1.05	0.38 → -0.5
**F/22**	No	ESL 1600, CLB 15, BRV 200	ESL 1600, CLB 15, BRV 200	FIAS	6	↓ ≥ 25 < 50%	12	-0.59 ↑ 0.53	-0.56 → -0.59	0.25 → 0.5
**M/50**	No	ESL 1600, LEV 1500, CLB 30	ESL 1600, LEV 1500, CLB 30	FIAS	4	↓ ≥ 75% < 90%	13	-1.63 → -3.14	-1.85 → -2.56	0.25 → 0.75
**M/39**	No	ZNS 500, LTG 500, CLB 50	ZNS 500, LTG 400 ↓, CLB 40 ↓	FIAS	0,5	↑ ≥ 75% < 90%	40	-3.97 → -7.32	-2.63 → -4.35	-1.63 → -1.75

**F/28**	No	ZNS 400, LEV 3000, OXC 1800	ZNS 400, LEV 3000, ECL 2000	FIAS	10	↓ ≥ 25% < 50%	72	NA → 0.28	0.15 → 0.45	-0.5 → -0.5
**M/32**	No	LCM 400, LTG 400, CBZ 800, CLB 20	LCM 400, LTG 400, CBZ 800, CLB 20	FIAS	10	↓ ≥ 25% < 50%	112	0.03 → 0.42	-1.87 → -0.51	-0.5 → -0.5
**F/38**	Yes/present	PER 6, CLB 20	PER 4 ↓, CLB 20	FBTCS	4	↓ < 25%	15	-7.04 → -2.32	-6.13 → -1.46	-1.13 → 0.5
**M/52**	No	ECL 2000, PGB 600	OXC 1200, PGB 450 ↓, LEV 1000 +	FBTCS	0.5	↓ 100%	39	-1.44 → -0.97	NA → -0.52	NA → 3.25

↓= decrease/reduction, ↑ = increase, + = add on, % = withdrawn. ASM, antiseizure medication; FAS, focal aware seizure; FBTCS, focal to bilateral tonic clonic seizure; FIAS, focal impaired awareness seizure; FLE, frontal lobe epilepsy; TLE, temporal lobe epilepsy; BRV, brivaracetam; CBZ, carbamazepine; CLB, clobazam; ESL, eslicarbazepine acetate; LEV, levetiracetam; LCM, lacosamide; LTG, lamotrigine; OXC, oxcarbamazepine; PGB, pregabalin; PER, perampanel; ZNS, zonisamide; LOCF, last observation carried forward. The green boxes in the 'ASMs at LOCF' column highlights ASM decrease/reduction, while the red boxes indicate ASM burden increase. In the 'Change in predominant seizure type from baseline' column, green highlights more than 50% seizure reduction, and red indicates more than 50% seizure increase. In TMT-A, TMT-B and DB change' column, green highlights patients with clinically significant improvement, while red signifies those with worsening.
